# Splenic Tuberculosis in a Patient With Crohn’s Disease Receiving Anti-tumor Necrosis Factor-α (TNF-α) Therapy: A Case Report

**DOI:** 10.7759/cureus.107723

**Published:** 2026-04-26

**Authors:** Abdelhalim Boucaid, Nabil Tiresse, Adil Zegmout, Hicham Souhi, Ismail Rhorfi, Hanane Elouazzani

**Affiliations:** 1 Department of Pulmonology, Mohammed V Military Training Hospital, Rabat, MAR; 2 Department of Phthisiology, Mohammed V Military Training Hospital, Rabat, MAR

**Keywords:** adalimumab, anti-tnf-α therapy, extrapulmonary tuberculosis, splenic tuberculosis, tuberculosis

## Abstract

Splenic tuberculosis is a rare manifestation of extrapulmonary tuberculosis, particularly presenting as isolated splenic disease. This study reports a 36-year-old man with Crohn's disease on adalimumab who presented with constitutional symptoms and multiple splenic nodules on imaging. Although initial latent tuberculosis screening using QuantiFERON in 2023 was negative, seroconversion was documented at admission. It should be noted that interferon-gamma release assays (IGRAs) (QuantiFERON) detect only *Mycobacterium tuberculosis* complex sensitization and do not detect non-tuberculous mycobacteria (NTM) or other bacterial pathogens; therefore, negative IGRA results do not exclude alternative mycobacterial or bacterial splenic disease. Image-guided splenic biopsy confirmed *Mycobacterium tuberculosis* with rifampicin susceptibility and caseating granulomas. Following adalimumab discontinuation and initiation of antituberculous therapy (isoniazid, rifampicin, pyrazinamide, and ethambutol {HRZE}), clinical remission was rapid and complete. This case emphasizes that splenic tuberculosis should remain in the differential diagnosis of patients receiving anti-tumor necrosis factor-α (TNF-α) agents, even after prior negative screening, and that early diagnosis via biopsy and rapid molecular testing (GeneXpert) significantly improves outcomes. Sustained vigilance and multidisciplinary coordination between gastroenterology, infectious diseases, and diagnostic imaging are essential in managing suspected tuberculosis in this high-risk population.

## Introduction

Tuberculosis (TB) remains a major global infectious disease and continues to manifest beyond the lungs, particularly in endemic settings [1]. Extrapulmonary TB, including abdominal involvement, represents an important clinical burden; abdominal TB may affect the gastrointestinal tract, peritoneum, lymph nodes, and solid organs such as the liver and spleen [1]. Within this spectrum, splenic TB is uncommon and is most often described in disseminated or miliary disease, typically among immunocompromised patients [2]. Yet, isolated or predominantly splenic TB has been increasingly recognized and remains diagnostically challenging, especially in immunocompetent individuals [3].

Patients frequently present with non-specific symptoms (e.g., constitutional symptoms, fever of unknown origin, or left upper quadrant pain), and imaging findings are often non-specific, which can lead to diagnostic delay and, at times, invasive investigations [1]. This challenge is particularly relevant in the biologic era. Anti-tumor necrosis factor-α (TNF-α) therapies used in Crohn's disease are associated with an increased risk of TB, including extrapulmonary and disseminated forms, despite recommended baseline screening strategies [4].

While *Mycobacterium tuberculosis* is the predominant mycobacterial pathogen affecting the spleen, other mycobacterial species, including non-tuberculous mycobacteria (NTM) such as *Mycobacterium avium* complex (MAC), may also present with splenic involvement, particularly in severely immunocompromised hosts [5]. The present case of splenic TB in an adalimumab-treated patient with prior negative latent TB screening illustrates this diagnostic dilemma and highlights the importance of maintaining a high index of suspicion, employing rapid molecular diagnostics, and coordinating multidisciplinary care in managing TB in immunosuppressed populations receiving anti-TNF-α therapy.

## Case presentation

A 36-year-old Moroccan man diagnosed with Crohn's disease in 2014 was admitted for evaluation of constitutional symptoms and splenic lesions in January 2026. He was a teacher. He had no known tuberculosis (TB) exposure and no personal history of active TB disease. He reported no toxic habits. The patient had received Bacillus Calmette-Guérin (BCG) vaccination and had no other comorbidities. Regarding inflammatory bowel disease therapy, he had initially been treated with azathioprine until 2024, after which he was switched to adalimumab (Humira). Adalimumab was administered according to the standard Crohn's disease regimen as follows: 160 mg subcutaneously on day one, 80 mg on day 15, followed by 40 mg every two weeks starting on day 29 for maintenance. The last adalimumab injection was administered in December 2025, and he was receiving concomitant corticosteroids (prednisone 10 mg/day). A latent TB screening performed in 2023 using an interferon-gamma release assay (QuantiFERON) had been negative; tuberculin skin test (TST) was not performed, and T-SPOT.TB was unavailable at that time.

The patient reported approximately 25 days of progressively worsening marked asthenia with functional limitation, associated with night sweats, anorexia, and an unintentional weight loss of ~6 kg over two months, evolving in an apyrexial context. He also complained of left upper quadrant (left hypochondrial) abdominal pain. He denied cough, dyspnea, chest pain, hemoptysis, and diarrhea.

On admission, the patient was alert and oriented, breathing comfortably on room air with an oxygen saturation of 98%. Vital signs were stable as follows: blood pressure 120/80 mmHg, heart rate 70 bpm, respiratory rate 16 breaths/min, and temperature 37°C. His weight was 50 kg and height 170 cm (BMI ≈ 17.3 kg/m²). Pulmonary examination showed symmetrical chest expansion with no crackles. Cardiovascular examination revealed a regular rhythm without abnormal findings. The abdomen was soft, with no hepatosplenomegaly. Peripheral lymph node examination was normal. There were no clinical signs of active Crohn's flare, and skin, joint, and ENT examinations were unremarkable.

**Figure 1:**
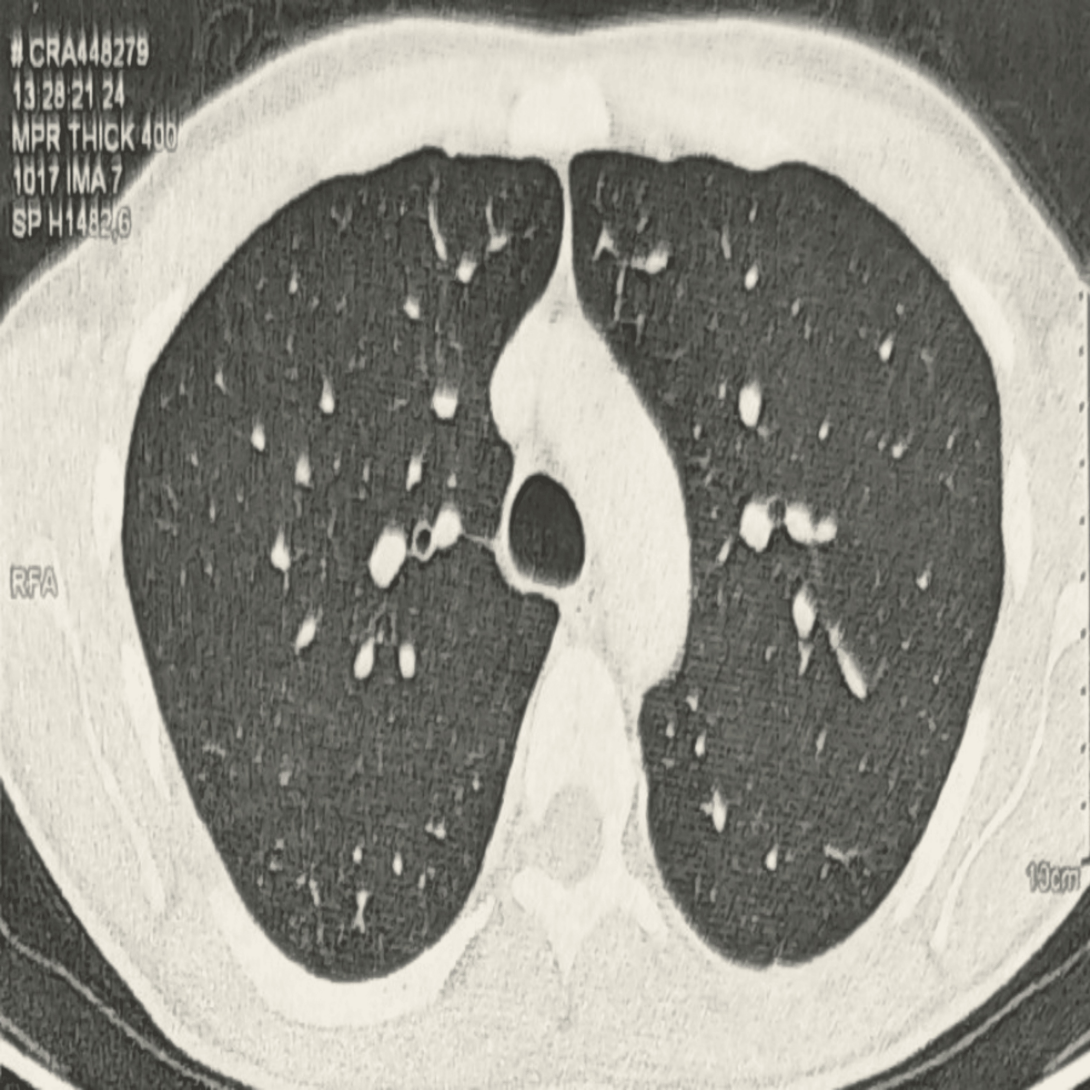
High-resolution axial thoracic CT demonstrates normal lung parenchyma bilaterally with no pleural effusion, mediastinal lymphadenopathy, or other abnormalities.

**Figure 2:**
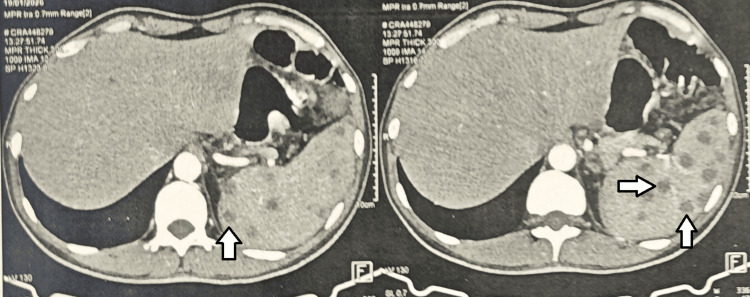
Axial abdominal CT demonstrates a normal-sized spleen containing multiple hypodense nodules measuring approximately 5-14 mm. The arrows highlight the focal splenic lesions corresponding to tuberculous involvement, helping to emphasize the abnormal areas of interest described in the radiological findings.

A contrast-enhanced thoracoabdominal CT scan performed on January 19, 2026, showed no pulmonary parenchymal lesions, no mediastinal or hilar lymphadenopathy, and no pleural or pericardial effusion (Figure 1). The spleen was normal in size and contained multiple hypodense nodules (white arrows) measuring approximately 5-14 mm, no intra- or retroperitoneal lymphadenopathy, and no peritoneal infiltration or effusion were identified (Figure 2).

**Figure 3:**
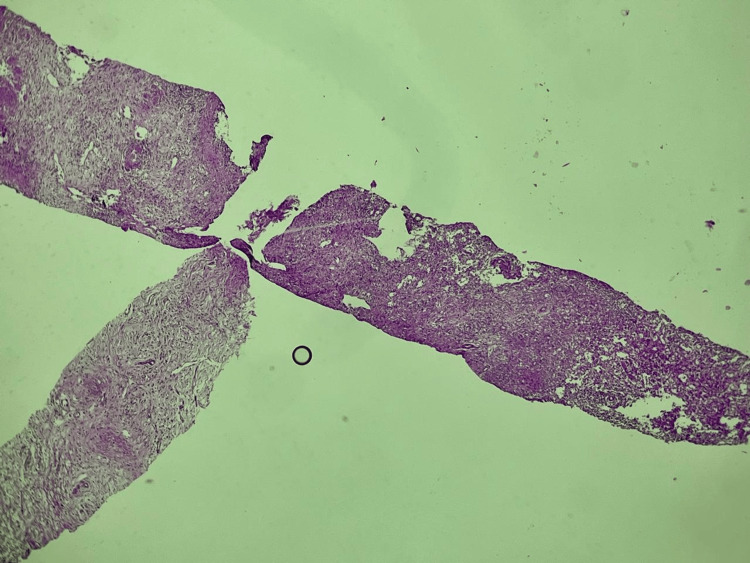
Low-power view showing splenic tissue extensively infiltrated by granulomatous inflammation with multiple coalescent nodules.

**Figure 4:**
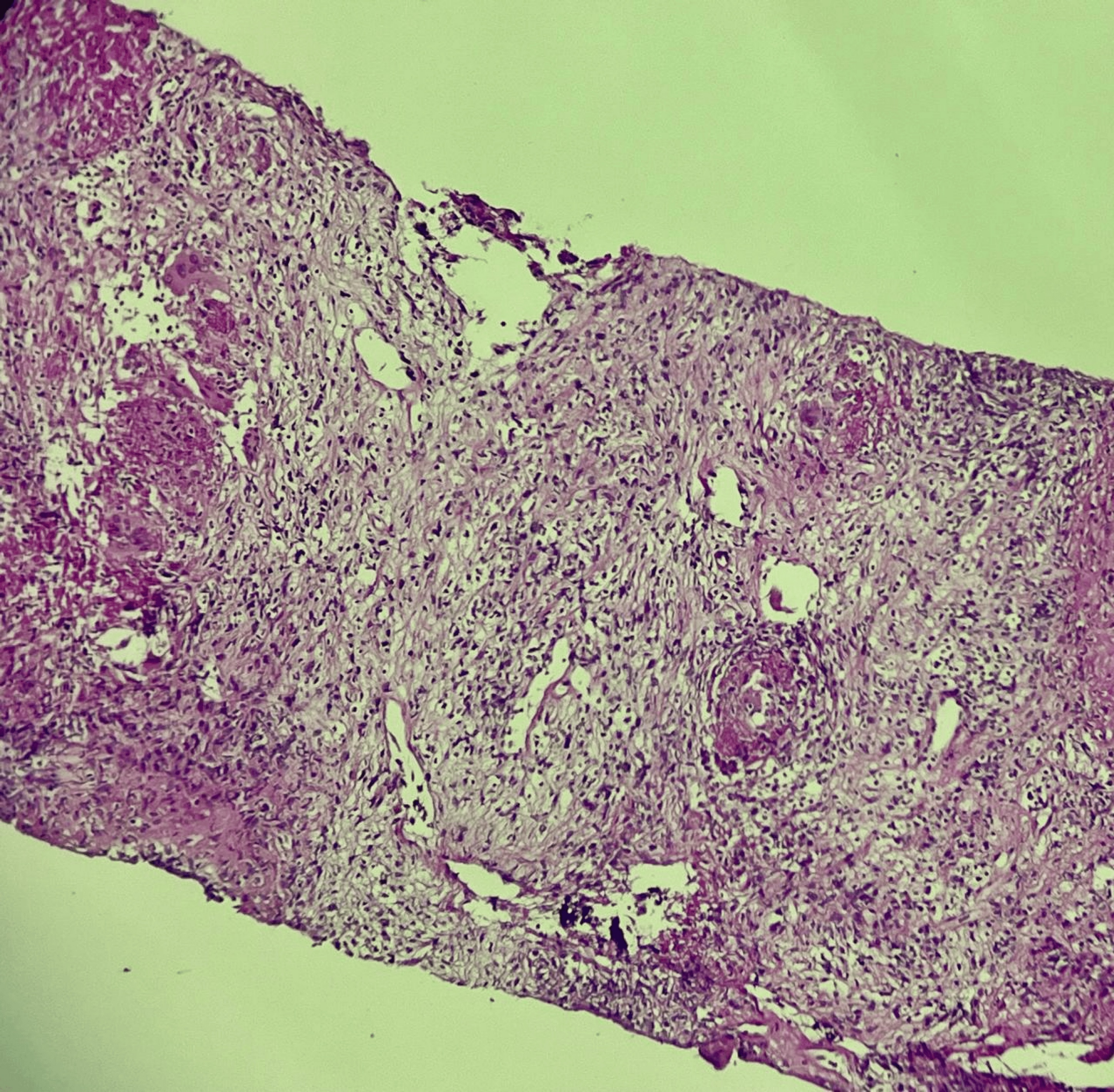
Higher magnification demonstrating epithelioid histiocytes, Langhans-type multinucleated giant cells, and surrounding lymphocytic infiltrate (H&E ×200).

Given the presence of a multinodular spleen, the initial differential diagnosis included infectious causes (tuberculosis, fungal infections such as candidiasis and histoplasmosis, pyogenic abscesses), hematologic malignancy (lymphoma), and metastatic disease. The final diagnosis was established by microbiological confirmation (GeneXpert) and supportive histopathology (Figures 3, 4).

Laboratory investigations showed an inflammatory syndrome with a C-reactive protein (CRP) of 65 mg/L and an elevated erythrocyte sedimentation rate (ESR). The white blood cell count was 5,800 cells/µL, and the platelet count was 170,000 cells/µL. Hemoglobin was 9.4 g/dL with an iron-deficiency profile characterized by microcytosis (low mean corpuscular volume {MCV}), low mean corpuscular hemoglobin {MCH}/mean corpuscular hemoglobin concentration (MCHC), and low ferritin, for which an iron supplementation was initiated. Liver function tests, renal function, serum albumin, and lactate dehydrogenase (LDH) were within normal limits. HIV, hepatitis B (HBV), and hepatitis C (HCV) serologies were negative. Immunologic testing for TB showed a QuantiFERON conversion from negative to positive compared with the prior negative result in 2023; however, this conversion cannot distinguish between TB reactivation and recent reinfection, nor does it confirm that the current infection originated from the same prior exposure.

Given the imaging findings of multinodular splenosis and QuantiFERON conversion in a patient receiving anti-TNF-α therapy, image-guided splenic biopsy was performed on January 22, 2026. GeneXpert *Mycobacterium tuberculosis* (MTB)/rifampicin (RIF) analysis of splenic tissue confirmed *Mycobacterium tuberculosis* with preserved rifampicin susceptibility. Mycobacterial culture subsequently yielded positive growth after two months of incubation, corroborating microbiological diagnosis. Respiratory investigations, including sputum PCR and mycobacterial culture, remained negative, excluding pulmonary disease. Histopathological examination revealed epithelioid-cell granulomas with multinucleated giant cells and caseous necrosis characteristic of tuberculosis, while excluding alternative diagnoses, including malignancy. A final diagnosis of isolated splenic tuberculosis was made in a patient with Crohn's disease receiving adalimumab.

Upon confirmation of active tuberculosis, adalimumab was discontinued. Standard first-line anti-tuberculous therapy (isoniazid, rifampicin, pyrazinamide, and ethambutol {HRZE}) was initiated at weight-based doses, isoniazid 5 mg/kg/day (maximum 300 mg/day), rifampicin 10 mg/kg/day (maximum 600 mg/day), pyrazinamide 25 mg/kg/day, and ethambutol 15 mg/kg/day, for a total duration of six months, following WHO 2023 and American Thoracic Society (ATS)/Infectious Diseases Society of America (IDSA) guidelines. Hepatotoxicity monitoring was performed weekly during the first month and monthly thereafter.

Clinical follow-up was scheduled every two weeks for the first two months, then monthly until completion of the six-month anti-tuberculous course. After one month of therapy, marked clinical improvement was evident with substantial symptom reduction and a 5 kg weight gain. Complete symptom resolution was documented by two months, treatment success was confirmed by complete resolution of constitutional symptoms, restoration of normal body weight through sustained weight gain, and complete radiologic clearance of splenic nodules on imaging at treatment completion. The gastroenterologist decided against reinitiation of adalimumab and opted for azathioprine as an alternative immunomodulatory agent for ongoing Crohn's disease management.

## Discussion

Splenic tuberculosis (TB) is an uncommon form of extrapulmonary TB and is particularly rare when it presents as isolated or predominant splenic disease in an immunocompromised host, which contributes to frequent diagnostic delay [1,2]. Accordingly, many patients present with non-specific constitutional symptoms and inconclusive routine laboratory findings, making early recognition challenging in daily practice [6]. The clinical spectrum is broad, ranging from fatigue, weight loss, splenomegaly, or pyrexia of unknown origin to more severe complications such as hypersplenism, portal hypertension, and splenic rupture [7,8].

Diagnostic uncertainty is often amplified by imaging because abdominal TB, including splenic involvement, may mimic a wide range of inflammatory and neoplastic conditions, requiring a high index of suspicion [2,9]. In addition, a multinodular spleen is not pathognomonic and overlaps with lymphoma, metastases, and infectious micro‑abscesses; splenic TB has repeatedly been reported as a “malignancy mimic,” including presentations initially interpreted as metastatic disease [9,10]. This overlap explains why malignancy was initially considered in our patient and underscores the need to pursue tissue diagnosis early when imaging is indeterminate [9].

In the present case, the clinical relevance is strengthened by exposure to anti‑TNF‑α therapy (adalimumab) for Crohn’s disease, as TNF blockade is a well‑established risk factor for TB, often with extrapulmonary or disseminated patterns, even after negative baseline screening [4,5]. Given the three-year interval between the initial negative QuantiFERON screening (2023) and symptom onset, recent TB reinfection remains a plausible possibility that cannot be formally excluded and warrants clinical consideration. In global safety data, roughly 62% of adalimumab‑associated TB cases are extrapulmonary and/or disseminated, consistent with TNF‑α blockade facilitating reactivation of secondary foci and subsequent mycobacterial spread [11].

Mechanistically, TNF‑α is pivotal for adhesion/chemokine signaling, macrophage antimicrobial effector functions and apoptosis, and for coordinated monocyte/T-cell recruitment and stable granuloma formation that contains infection [12-14]. Because infliximab and adalimumab bind TNF‑α (including transmembrane TNF) with high affinity, they may promote macrophage/T‑cell apoptosis and blunt T‑cell activation and interferon-gamma (IFN‑γ) production, thereby weakening host defense against *Mycobacterium tuberculosis* [15-19].

Laboratory data are frequently abnormal but rarely diagnostic in isolation; hematologic abnormalities can mimic hematologic malignancies, and inflammatory markers are non-specific [8]. In our patient, the lack of hypersplenism and the absence of leukopenia/thrombocytopenia did not exclude splenic TB, reinforcing that laboratory findings should be interpreted alongside clinical context and imaging [6,8].

Given these limitations, tissue confirmation remains essential; compared with cases requiring diagnostic splenectomy, an image‑guided splenic biopsy with rapid molecular testing (GeneXpert/NAAT) aligns with published experience showing that PCR‑based methods can secure diagnosis when imaging is misleading [1,2,6]. Immunologic tests may be repeated during anti‑TNF therapy in order to detect possible interferon-gamma release assay (IGRA) conversions, supporting vigilance even after initially negative screening [20].

Finally, management is consistent with regulatory guidance recommending interruption of anti‑TNF therapy when TB is suspected or confirmed, followed by full antituberculous treatment and close monitoring. The favorable clinical and radiologic response in our patient underscores the benefit of early diagnosis and timely therapy.

## Conclusions

Splenic tuberculosis is a rare form of extrapulmonary tuberculosis presenting with non-specific symptoms, often causing diagnostic delay. In the present case of a patient with Crohn's disease receiving adalimumab who presented with multinodular splenic disease, tuberculosis remained an important differential diagnosis. Acute TB infection was definitively established through culture and PCR identification of *Mycobacterium tuberculosis* complex from splenic tissue.

Because imaging findings can mimic malignancy, early tissue confirmation using image-guided biopsy and rapid molecular testing proved crucial in avoiding unnecessary surgery and enabling timely treatment initiation. In this patient, discontinuation of adalimumab combined with standard antituberculous therapy (HRZE for two months followed by HR for four months) resulted in marked clinical improvement within one month, complete symptom resolution by two months, and complete radiologic resolution at treatment completion. The patient remains under ongoing clinical surveillance. This case demonstrates the importance of maintaining a high index of suspicion for tuberculosis in patients receiving anti-TNF-α therapy, employing rapid microbiological diagnosis, and implementing coordinated multidisciplinary management to achieve favorable outcomes.
